# Electrophysiological recordings reveal photoreceptor coupling in the dorsal rim areas of honeybee and bumblebee eyes

**DOI:** 10.1098/rsbl.2025.0234

**Published:** 2025-09-03

**Authors:** George E. Kolyfetis, Gregor Belušič, James J. Foster

**Affiliations:** ^1^ Department of Biology, University of Konstanz, Konstanz, Germany; ^2^ Centre for the Advanced Study of Collective Behaviour, University of Konstanz, Konstanz, Germany; ^3^ International Max Planck Research School for Quantitative Behaviour, Ecology and Evolution(IMPRS/QBEE), Max Planck Institute of Animal Behavior, Radolfzell 78315, Germany; ^4^ Department of Biology, Biotechnical faculty, University of Ljubljana, Ljubljana, Slovenia

**Keywords:** vision, navigation, polarization, bees

## Abstract

Many insects rely on skylight polarization patterns to navigate their habitats. To perform this vital task, most insect species have evolved specialized ommatidia in the dorsal rim area (DRA) of their compound eyes, which are adapted to detect linearly polarized light in large patches of the sky. In this study, we conducted electrophysiological recordings of ultraviolet-sensitive photoreceptors in the DRA and other regions of the compound eyes in honeybees (*Apis mellifera*) and bumblebees (*Bombus terrestris*) to map their receptive fields (RFs). For both species, we report novel evidence for photoreceptor coupling, i.e. spatial summation, recorded in the retinal layer of the DRA. We explore spatial summation as a possible mechanism to increase the effective size of DRA ommatidia RFs, a crucial functional feature of the polarization compass.

## 1. Introduction

Light arriving from the Sun gets scattered and polarized in the Earth’s atmosphere, creating patterns of polarization in the sky [1,2]. These patterns depend on the Sun’s position and can thus act as a valuable navigational reference system for many insects that perceive them [2–4]. Honeybees have been behaviourally demonstrated to utilize an internal map of skylight polarization’s topography, a ‘polarization compass’, to estimate the position of the Sun and navigate successfully [5,6].

Most insect species detect polarized light using specialized, polarization-sensitive photoreceptors in the dorsal rim area (DRA) of their compound eyes [7–9]. In honeybees and bumblebees, these are UV-sensitive photoreceptors with high polarization sensitivity (PS) [7,10–12]. Honeybee DRA photoreceptors also possess exceptionally wide receptive fields (RFs) compared with the main retina and dorsal-eye photoreceptors [7]. The shape of these wide RFs has been previously described in honeybees; they exhibit a central region with high relative sensitivity and a wide ‘brim region’ surrounding the centre, where sensitivity decreases very gradually with increasing off-axis angle [7].

Little is known about the mechanism underlying the wide DRA RFs. It has been proposed that light-scattering structures, such as rugged-walled pore canals, in the corneal lenses of the DRA ommatidia of several hymenopteran species [13] could result in increased sensitivity to off-axis illumination, and thus extended RFs [7,14]. The existence of pore canals or wide RFs in the bumblebee DRA remains unknown. For crickets, both anatomical (lack of screening pigment and corneal facets; [15]) and neural (spatial integration via polarization-sensitive interneurons; [16,17]) adaptations have been proposed as the source of wide DRA RFs. Neural spatial integration of this type, acting as a spatial low-pass filter, can smooth out local disturbances in the skylight polarization pattern (e.g. due to clouds), increase the polarization signal-to-noise ratio, and thus aid navigational efficiency [16]. The wide RFs of DRA photoreceptors naturally result in only low spatial resolution, making them relatively insensitive to small local changes in brightness or polarization. They have, however, been reported to have high absolute sensitivity, in terms of photon capture [15,18], which would enhance their capacity to detect small changes across the RF as a whole.

In this study, we mapped the RFs of honeybee (*Apis mellifera*) and bumblebee (*Bombus terrestris*) DRA photoreceptors by recording from them electrophysiologically. We identified photoreceptor coupling from recordings at the retinal layer in both species’ DRAs, which constitutes novel evidence of eye-region-specific neural spatial summation present in signals from the retinal layer. We discuss cell coupling as a potential, plastic mechanism for increasing the effective size of bee DRA RFs, and, in light of this new discovery, we reexamine the function of pore canals in polarization vision.

## 2. Methods

### (a) Electrophysiological photoreceptor recordings

**Figure 1.**
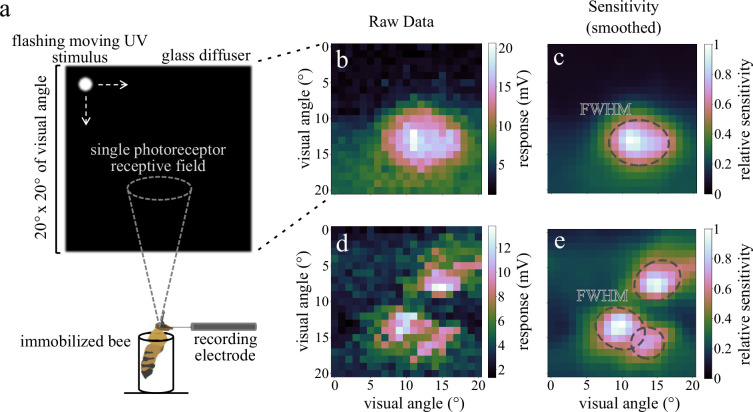
Experimental set-up and receptive field (RF) mapping. (a) Overview of the experimental set-up. Bees were immobilized and presented with a flashing, moving UV stimulus while we recorded responses from single photoreceptors. (b) Raw mV and sensitivity-transformed (smoothed) (c) response output of a non-coupled photoreceptor. (d) Raw mV and sensitivity-transformed (smoothed) (e) response output of a coupled DRA photoreceptor. Dotted lines outline the full width at half maximum (FWHM) of the RFs. These spatial sensitivity recordings allowed us to map individual and coupled RFs.

We immobilized honeybee (*Apis mellifera mellifera* and *Apis m. carnica*) and bumblebee (*Bombus terrestris dalmatinus*) workers in plastic pipette tips using beeswax and resin, and carefully aligned the head with the stimulus. For main retina and DRA recordings, a microelectrode (0.5/1.0 mm inner/outer diameter borosilicate glass, filled with 3M KCl, resistance *R *> 100 MΩ) was inserted into the cornea ventral to the DRA, through a small hole (figure 1a), connected to an amplifier (SEC10LX, NPI electronic, Germany) and advanced with a piezo micromanipulator (Sensapex, Finland). The experiments were conducted in a dark laboratory, in a Faraday cage made of iron plates, using dense stimulation with bright light flashes, so that the animals were not dark-adapted.

All cells were initially stimulated with a multispectral light synthesizer (LED synth) [19] for 2 s to assess their spectral sensitivity. We fitted a visual pigment template function to the spectral responses [20]. PS was evaluated with a flash stimulus from a Xe arc lamp and a monochromator (B&M Optik, Germany), projected through a polarizer (OUV2500, Knight Optical, UK) at the peak wavelength of the determined spectral sensitivity. Then, unpolarized flashes at the same wavelength, projected through a graded ND filter (max. UV intensity approx. 10^14^ photons cm^−2^ s^−1^), were used to measure the intensity–response function (‘V-log(I)’). Single-cell responses were nonlinearly transformed into sensitivity values using the cell’s intensity response function and an inverse Hill transformation [21]. We then calculated PS as the ratio between maximum and minimum sensitivity to light, polarized at different angles [22]. Where multiple polarization responses were measured for one cell, a per-angle arithmetic mean of the raw measurements was calculated first. Mean PS values above 20 were considered artefactual owing to the low or even hyperpolarizing minimum response to polarized light measured (possibly due to inhibition from polarization-opponent photoreceptors) [23] and were thus excluded.

We mapped spatial RFs of UV-sensitive cells by presenting flashes from a 365 nm UV LED (Cairn, UK) at different positions on a rear-illuminated, UV-transmissive glass diffuser using a pair of AT20L-L galvanometer mirrors (Shenzhen City Aoxinjie Technology, China). The centre of the photoreceptor RF was determined by using moving horizontal and vertical bars, and the whole RF was mapped with a bright, flashing 0.9° × 0.9° square stimulus. The stimulus was controlled from the voltage command output of the recording software (WinWCP [24]): two analogue outputs delivered voltage steps to move the two galvanometer mirrors, while a digital output delivered a 20 ms pulse every 145 ms to turn on the UV LED in 1° steps, moving left to right, top to bottom on a 20° × 20° grid (figure 1a).

PS was re-measured in some coupled DRA cells by aligning their multiple optical axes with the stimulus and measuring PS in the different parts of their RF.

For further recording details, see electronic supplementary material, methods.

### (b) Receptive field modelling

**Figure 2.**
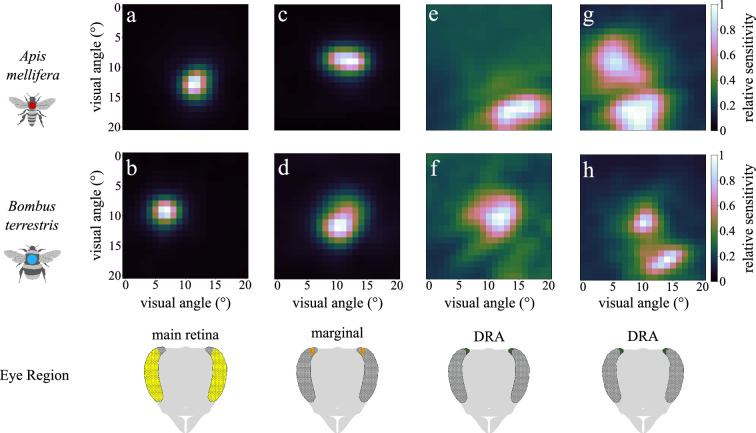
Receptive field (RF) shapes in different eye regions of honeybees and bumblebees. Main retina (a,b), marginal DRA (c,d), non-coupled DRA (e,f) and coupled DRA (g,h) photoreceptor RF shapes for honeybee (*A. mellifera*) and bumblebee (*B. terrestris*), respectively. In both species, RF size increases from the main retina to the DRA, and their shapes become more irregular. Coupled DRA photoreceptor RFs were observed in both species.

To model RFs of main retina and DRA UV-sensitive cells (16 honeybee cells and 31 bumblebee cells in total), we divided every row of the 20° × 20° raw spatial sensitivity (SS) data grid into 21 equal parts, extracting the maximum voltage response values for each. From the maximum values, we subtracted the minimum response of each part, which roughly corresponds to the background noise. These modified raw SS data (figure 1b,d) were smoothed using Gaussian filtering (scipy.ndimage.gaussian_filter; sigma = 1°, all other parameters set to default) to acquire smoothed SS data. We then applied the inverse Hill function to normalized SS datasets to acquire final SS data (figures 1c,e and 2).

RF size was quantified by fitting two-dimensional, elliptical Gaussians. For coupled photoreceptors, we added a ‘weighting’ parameter to each RF, such that the RF with the largest amplitude (greatest contribution to modelled response density) received the strongest weighting. This allowed us to distinguish the putative main cell (punctured; high weighting) from the coupled one(s) (likely distant from the electrode tip; low weighting). Only the main cell properties were included in the summary analyses. Recorded cells were categorized into main retina, marginal DRA and DRA based mainly on the recording depth (topologically), but also their intensity–response dynamics (main retina and marginal DRA: high sensitivity; DRA: low sensitivity), PS (main retina: low PS, marginal and DRA: high PS) and approximate size of their RFs. Where we had multiple SS recordings for a single cell, an average circular full width at half maximum ((FWHM) of high-quality recordings) was calculated.

For further RF modelling details, see electronic supplementary material, methods.

### (c) Statistical analyses and visualizations

**Figure 3.**
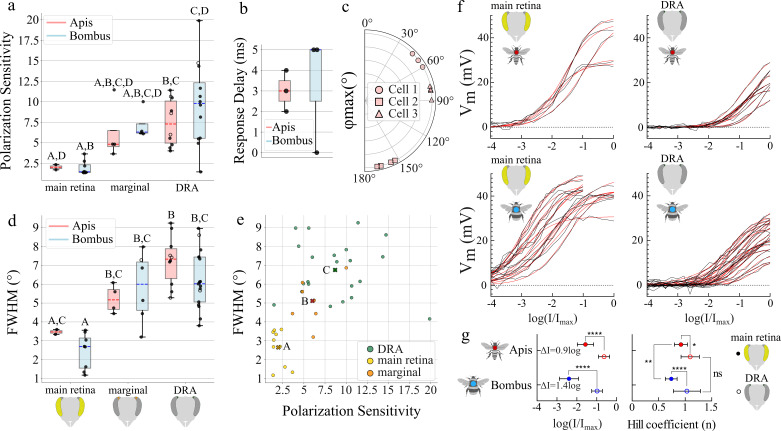
Overview of visual properties in the different eye regions. (a) Polarization sensitivity in the different eye regions. (b) Response delay differences between coupled cells (*t*_max_main_cell_ − *t*_max_secondary_cell_). (c) Comparisons of the preferred angle of polarization (*φ*_max_) for different receptive fields (RFs) from the same cell, for three honeybee DRA photoreceptor cells. (d) RF size (represented by their FWHM) in the different eye regions. (e) Relationship between RF size (FWHM) and PS in the different eye regions. Crosses indicate the grand means for each eye region; letters indicate significant differences. (f) Intensity–response curves of UV receptors in different eye regions (black curves) in both species. (g) Left: half-maximum intensity parameters of Hill functions (red curves in (e)), fitted to voltage data; right: Hill slope parameters of Hill functions. Whiskers in (a) and (b) represent Q1 − 1.5 × IQR (lower whisker) and Q3 + 1.5 × IQR (upper whisker). Different letters indicate significant differences. Open circles indicate coupled cells (a,d).

A two-way ANOVA was performed on PS and FWHM data (figure 3a,d). PS and FWHM residuals were normally distributed (Shapiro–Wilk *p* = 0.2093 and *p* = 0.9255, respectively). Tukey’s HSD *post hoc* tests were carried out on both PS and FWHM data.

A linear model accounting for differences between eye regions and species was fitted to the FWHM ~ PS data (electronic supplementary material, table S1) after model selection. Residuals were normally distributed (Shapiro–Wilk *p* = 0.5282), and a Tukey’s HSD *post hoc* test was performed.

Statistical analyses were performed in Python using the scipy and statsmodels packages. Figure visualizations were performed using Python’s Matplotlib [25] and Inkscape [26].

## 3. Results

We performed electrophysiological recordings of 17 honeybee (*A. mellifera*; *n* = 9 individuals) and 31 bumblebee (*B. terrestris*; *n* = 8 individuals) UV-sensitive photoreceptors in the main retina (*n* = 10), marginal DRA (*n* = 11) and DRA (*n* = 27) to assess spatial, spectral and polarization sensitivity (PS). We also recorded from blue-sensitive photoreceptors in the main retina, but did not encounter any in the DRA, where they are thought to be absent [7,12]. Honeybees’ and bumblebees’ peak wavelength sensitivity of UV photoreceptors lies at approximately 340 nm, similar to previous reports [7,12].

DRA photoreceptors have the largest RFs across all eye regions in both species (figures 2 and 3d). Furthermore, in six recordings (three per species), DRA cells exhibited multiple regions of high relative sensitivity (>50% of maximum) that were separated by low-sensitivity regions (usually <20% of maximum). These additional secondary RFs likely belong to neighbouring, coupled photoreceptor cells that only partly overlap in their visual fields but share their responses. Examples of this photoreceptor coupling in the DRA appear in figures 1d,e and 2g,h. Analysis of maximum-response delay differences (*Apis*: 2, 3 and 4 ms; *Bombus*: 0, 5 and 5 ms; figure 3b) and PS angular maxima between primary and secondary RFs (figure 3c) confirmed that the multiple RFs were not the result of optical artefacts or of multiple-cell electrode puncturing (see also electronic supplementary material).

RF size differences across eye regions are shown in figure 2. Main retina RFs are small, circular and radially symmetric with high sensitivity at the centre, which decreases rapidly at distances of 1°–2°. Marginal DRA RFs exhibit similar characteristics but have a more elliptical shape and are slightly larger compared with the main retina. DRA photoreceptors’ RFs cover the largest visual angle. They have irregular shapes and can be characterized by wide regions of low, non-zero sensitivity around their centres. Their SS decreases more gradually from the centre of the RFs than the sensitivity of marginal and main retina cells (figures 1b–e and 2e–h). Coupled photoreceptors cover an even wider area of the total visual scene, combining RFs of different cells.

An overview of UV photoreceptor properties in different eye regions can be seen in figure 3. *Apis* DRA mean PS (7.48) was significantly higher than *Apis* mean main retina PS (2.02) (*p* = 0.0222). *Bombus* DRA mean PS (9.71) was also significantly higher than *Bombus* mean main retina PS (1.95) (*p* = 3 × 10^−4^). For marginal DRA cells, a mean PS of 6.19 was determined for honeybees and 7.18 for bumblebees (figure 3a).

In the main retina, UV and blue receptors had their angle of maximal PS (*φ*_max_) aligned with the dorsoventral eye axis. In contrast, DRA UV receptors showed *φ*_max_ orientations loosely parallel or perpendicular to the eye’s edge. In coupled DRA receptors, the *φ*_max_ of the coupled cells was offset by 3°–5° from the *φ*_max_ of the main cell (figure 3c; electronic supplementary material, figure S3), indicating coupling between homologous cells, arranged in a fan shape, typical for the DRA [27].

For ease of comparison, all elliptical Gaussian RFs were converted to circular RFs of equal area, and we report their FWHM. *Apis* DRA mean RF (7.24°) was significantly larger than *Apis* and *Bombus* mean main retina RF (3.47°, 2.45°; *p* = 0.0113 and *p* < 1 × 10^−4^, respectively). *Bombus* DRA mean RF (6.23°) was significantly larger than *Bombus* mean main retina RF (*p* < 1 × 10^−4^). Furthermore, *Bombus* mean main retina RF was significantly smaller than *Apis* and *Bombus* marginal DRA RF (5.22°, 5.82°; *p* = 0.0226 and *p* = 6 × 10^−4^, respectively; figures 2 and 3d).

Cells with higher PS also had larger RFs, both also differing between eye regions (figure 3e). After fitting a linear model accounting for species and eye region to the FWHM–PS data (electronic supplementary material, table S1), neither PS (*β* = 0.170°/PS, *p* = 0.799) nor species (*β* = −0.8793°, *p* = 0.062) significantly predicted the FWHM size. The model revealed significant differences between the main retina RF size and marginal DRA (*β* = 2.1868°, *p* = 0.004) and DRA (*β* = 3.7542°, *p* < 1 × 10^−4^) and a *post hoc* Tukey’s HSD test confirmed the differences. Main retina cells had significantly smaller RF size than both marginal DRA and DRA (mean diff = 2.46°, *p* = 0.0018 and mean diff = 4.09°, *p* < 1 × 10^−4^, respectively), and marginal DRA cells had significantly smaller RF size than DRA cells (mean diff = 1.64, *p* = 0.0198).

The cells also had strikingly different absolute light sensitivities: UV receptors in the main retina and marginal DRA were approximately 10 times more sensitive (honeybees: 0.9 log units; bumblebees: 1.4 log units) than their DRA counterparts (figure 3f,g). Finally, DRA photoreceptors of both species exhibited, on average, steeper Hill curve slopes (*n*) than main retina ones (honeybee: *n*_DRA_ = 1.095 versus *n*_main_ = 0.92, *p* = 0.0453; bumblebee: *n*_DRA_ = 1.033 versus *n*_main_ = 0.737, *p* < 1 × 10^−4^; figure 3g).

## 4. Discussion

In this study, we identify a novel form of retina-level spatial summation, exclusive to the DRA and adjacent marginal region. While our findings otherwise confirm the previously reported properties of honeybee (*A. mellifera*) and bumblebee (*B. terrestris*) DRA photoreceptors, this discovery reveals a potentially unique solution to the trade-off between PS and absolute sensitivity [28]. Around a quarter of our single-cell recordings in the DRA in both species showed multiple high-sensitivity regions separated by areas of low sensitivity, suggesting photoreceptor coupling. We are unable to discern whether these cells represent a subset of DRA rhabdoms in which coupling occurs, or if secondary RFs for other DRA cells were either too weak or too distant from the primary RF to be detected using our spatial-sensitivity stimulus. This coupling could act as a flexible mechanism of neural spatial summation that can be varied depending on the visual environment [29], allowing the visual system to balance the increase in sensitivity against the decrease in resolving power; a trade-off inherent in pooling signals from neighbouring units [25]. Indeed, photoreceptor coupling was not observed in our recordings from the main retina high-acuity zones adapted for object vision [30], where low spatial resolution would be maladaptive. Although differential resolution and sensitivity across apposition eye regions are possible through varying optical parameters [31], a neural mechanism permits rapid activation at low resource cost, requiring no extra cornea material. Numerous species enhance sensitivity under low-light or low-contrast conditions via neural spatial summation in the peripheral visual system, as found in the ganglion and bipolar cells in the vertebrate retina [32–34] and laminar monopolar cells in hawkmoths [35]. In the *Drosophila* DRA, cell coupling takes place in the medulla, where approximately 10 photoreceptors converge to one distal medulla DRA neuron (MEDRA) [36]. As previously reported for bumblebees [12], butterflies [37] and fruit flies [38,39], we also observed hyperpolarizations triggered by opponent interactions between photoreceptors within the same rhabdom. However, spatial summation in the responses of cells in separate ommatidia in the DRA retina has not been reported before. Since the primary function of the DRA is to distinguish changes in the polarization pattern in large sky patches [6,40], photoreceptor coupling could eliminate cloud noise by smoothing out the polarization pattern, as proposed for polarization-sensitive interneuron coupling in the cricket DRA [17]. At present, the pathways responsible for this coupling remain unidentified. It is possible that the the retinotopic organization of the DRA is mirrored in the distal medulla (MEDRA) [39,41], where connections between cartridges occur in *Drosophila*. In the blowfly *Calliphora erythrocephala*, specialized connectivity has been identified in the ommatidia in the outermost two rows along the rim of the eye [42]. For these ommatidia, there is no glial separation between photoreceptors from rhabdoms in different rows, forming a ‘double-pseudocartridge’ that would allow for electrical coupling between adjacent rhabdoms.

We find that UV-sensitive DRA photoreceptors in both bee species possess wide RFs and high PS. These large, overlapping RFs would capture a wide sky area, likely increasing signal and making navigation more efficient [7,16]. Rugged-walled pore canals in the DRA have been proposed to widen the ommatidial RFs by scattering incoming light [7,13]. Although we do not present any direct evidence in favour of this phenomenon here, we propose spatial summation via photoreceptor coupling as another cause of the wide DRA RFs. Pore canals could act as an aperture-limiter for the DRA ommatidia, scattering a portion of incident light out of the light path, reducing the quantity that reaches the rhabdoms. Since DRA ommatidia usually view the bright sky, this mechanism could prevent the otherwise rapid saturation of their photoreceptors. Indeed, the DRA intensity–response curves generally reached maximal response over a narrower range (larger slope parameter) than in the main retina (figure 3g). However, these photoreceptors showed lower sensitivity. This would allow the DRA receptors to better encode the small contrast changes that would be induced by changes in skylight polarization, while avoiding saturation at typical skylight intensities. Spatial summation via photoreceptor coupling may help compensate for this low sensitivity under low-contrast conditions. Since our stimuli were bright, sub-saturating flashes, the photoreceptors were not dark-adapted. It appears that this spatial summation mechanism is active under daylight conditions.

The observed DRA photoreceptor coupling could also potentially explain the wide low-sensitivity areas of the SS curve [7] previously attributed to the pore canals [12]. Contributions from coupled photoreceptors’ RFs would have been difficult to identify in previous studies, since the stimuli presented spanned only two orthogonal axes. However, it is possible that the two mechanisms act complementarily, pore canals widening each ommatidium’s RF and cell coupling expanding each photoreceptor’s effective RF [7] (figure 2e–h). The wide low-sensitivity areas (brims) of the SS curve described by Labhart [7] may be captured by the ‘offset’ parameter of our Gaussian models of the DRA RFs (see Methods). Future work focused on the function of the pore canals will be crucial to elucidate their role in insect polarization vision.

## 5. Conclusion

The results of this work provide the first evidence for photoreceptor coupling between adjacent rhabdoms in the DRAs of honeybee (*A. mellifera*) and bumblebee (*B. terrestris*) eyes. Our electrophysiological recordings show that DRA photoreceptors exhibit multiple RFs separated by regions of low SS. Response delay differences and slight changes in *φ*_max_ angles further corroborate the existence of photoreceptor coupling in the DRA. Importantly, spatial summation in the DRA may serve a crucial functional role, reducing noise in the detection of skylight polarization, and thus aiding navigation in bees. Overall, this study contributes valuable insights into bees’ visual systems and adaptations of the DRA for polarization vision.

## Data Availability

All data files needed to evaluate the conclusions in the article have been uploaded to Figshare [43]. Custom scripts used for analyses, statistical tests and visualizations have been uploaded to Figshare [43] and can also be found in the GitHub repository [44]. Supplementary material is available online [45].
